# Polyunsaturated Fatty Acids and Their Derivatives: Therapeutic Value for Inflammatory, Functional Gastrointestinal Disorders, and Colorectal Cancer

**DOI:** 10.3389/fphar.2016.00459

**Published:** 2016-12-01

**Authors:** Arkadiusz Michalak, Paula Mosińska, Jakub Fichna

**Affiliations:** Department of Biochemistry, Faculty of Medicine, Medical University of LodzLodz, Poland

**Keywords:** n-3 PUFA, n-6 PUFA, eicosapentaenoic acid, docosahexaenoic acid, arachidonic acid derivatives, inflammatory bowel disease, irritable bowel syndrome, colorectal cancer

## Abstract

Polyunsaturated fatty acids (PUFAs) are bioactive lipids which modulate inflammation and immunity. They gained recognition in nutritional therapy and are recommended dietary supplements. There is a growing body of evidence suggesting the usefulness of PUFAs in active therapy of various gastrointestinal (GI) diseases. In this review we briefly cover the systematics of PUFAs and their metabolites, and elaborate on their possible use in inflammatory bowel disease (IBD), functional gastrointestinal disorders (FGIDs) with focus on irritable bowel syndrome (IBS), and colorectal cancer (CRC). Each section describes the latest findings from *in vitro* and *in vivo* studies, with reports of clinical interventions when available.

## Introduction

Gastroenterology is a rapidly-evolving basic and clinical science that concerns organic and functional gastrointestinal (GI) disorders. The former include inflammatory, infectious and neoplastic diseases and the latter embrace conditions characterized by chronic symptoms and the absence of recognized biochemical or structural explanations.

Inflammatory bowel disease (IBD) has been constricted so far mainly to Europe and US, but currently emerge also in newly industrialized countries in Asia, Middle East, and South America (Kaplan, 2015). North America and Europe also remain the leading regions of the occurrence of functional gastrointestinal disorders (FGIDs), especially irritable bowel syndrome (IBS) (Fox and Muniraj, 2016). In turn, the incidence and mortality of colorectal cancer (CRC) are high in developed countries and are rapidly rising in low-income and middle-income populations (Arnold et al., 2016). Despite an ongoing progress in implementing structured screening in reducing the prevalence rate and developing new treatments, currently available options are not always effective and often prompt adverse effects (Favoriti et al., 2016). Hence, we still need new tools of prevention and therapy.

The aim of this review is to explore the studies on polyunsaturated fatty acids (PUFAs), their metabolites and derivatives, and relate the findings to the pressing problems of gastroenterology. The scientific bibliography included in this review was collected from the PubMed and Web of Science databases. We searched for English language review articles or original research published up to May 2016, using the following keywords alone or in combination: PUFA, arachidonic acid, linoleic acid, eicosapentaenoic acid, docosahexaenoic acid, fish oil, diet in irritable bowel syndrome, inflammatory bowel disease, Crohn's disease, ulcerative colitis, colorectal cancer, functional gastrointestinal diseases. Clinical trials were searched using ClinicalTrials.gov database.

## Systematic overview of PUFAs

A fatty acid (FA) comprises aliphatic hydrocarbon chain with methyl and carboxyl groups at opposite ends; PUFAs contain more than one double bond in their structure. There are two main groups of biologically important long chain PUFAs: n-6 PUFA with their first double bond at C6, counting from the methyl C, and n-3 PUFAs with first unsaturated bond at C3. The main representatives of these groups are:

- n-6 PUFAs: linoleic acid (LA, 18:2), arachidonic acid (AA, 20:4),
- n-3 PUFAs: alpha-linolenic acid (ALA, 18:3), eicosapentaenoic acid (EPA, 19:5) and docosahexaenoic acid (DHA, 22:6).

LA and ALA are referred to as essential FAs, because human body lacks the enzymes for their synthesis. Both LA and ALA are the precursors for AA, EPA and DHA, and are of primal biological importance—both FAs must be thus additionally supplemented in case of enzymatic defects or lack of dietary ALA or LA. In humans, diet remains a primary source of DHA and EPA because the efficacy of transforming ALA to longer n-3 PUFAs is low and personally variable (Glaser et al., 2010). EPA and DHA are mostly found in fish, especially salmon, whereas the most important source of ALA are seed oils derived from walnuts, chia, perilla, rapeseeds or soybeans. Currently, due to the suboptimal total PUFAs consumption in most populations, there is a growing awareness and support for regular PUFA supplementation (González-Rodríguez et al., 2013; Sioen et al., 2013; Kodentsova et al., 2014).

Physiologically, PUFAs are built into lipid membranes. Once they are released by phospholipase A2 (PLA2), they undergo processing to many biologically active signaling molecules (Gomolka et al., 2011; Maskrey et al., 2013; Capra et al., 2015; Powell and Rokach, 2015). The most important pathways of PUFAs metabolism include:

- cyclooxygenases (COXs)–act on both n-3 and n-6 PUFAs, yielding prostaglandins (PGs), prostacyclins and thromboxanes (TXs); altogether branded prostanoids,
- lipooxygenases (LOXs)–convert AA to lipoxins (LXs) and leukotrienes (LTs). Together with COXs, LOXs also produce protectins, marensins, and resolvins (Rvs) from n-3 PUFAs,
- cytochrome 450 (Cyp 450)–catalyzes the conversion of both n-3 and n-6 PUFAs to epoxyeicosatrienoic acids (EETs). Along with other enzymes, Cyp450 takes part in the synthesis of PUFA derivatives, producing biologically active hydroxyeicosatetraenoic acids (HETEs).

Polyunsaturated fatty acids (PUFAs) can also be processed by non-enzymatic oxidation, creating isoprostanes, and isofurans which also display biological activity (Galano et al., 2015; Roy et al., 2016).

**Figure 1:**
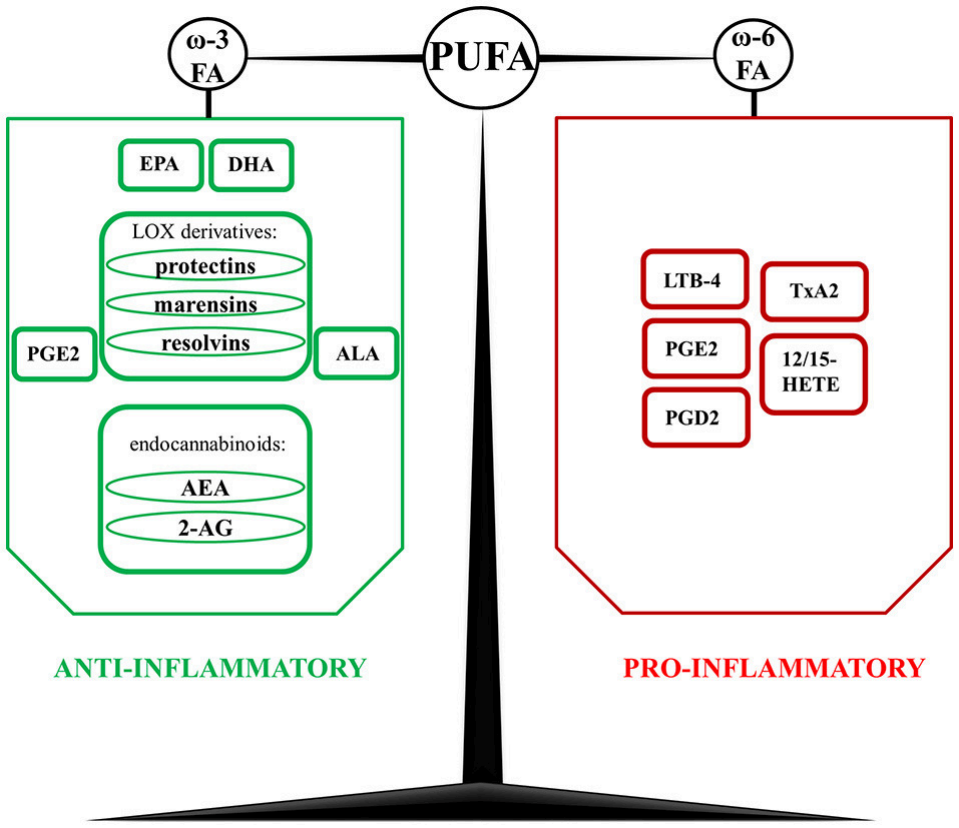
**Derivatives of polyunsaturated fatty acids (PUFA) with anti- and pro-inflammatory properties**. In contrast to n-3 PUFA-derived mediators, which are mostly considered as anti-inflammatory, n-6 PUFA derivatives promote inflammatory response. However, one of the unique metabolites of n-6 PUFA are lipoxins (Lx)- LxB4 and LxB5, which display a wide spectrum of anti-inflammatory and pro-resolution actions. AA, arachidonic acid; AEA, N-arachidonoylethanolamide, anandamide; ALA, alpha-linoleic acid; 2-AG, 2-arachidonoylglycerol; DHA, docosahexaenoic acid; EPA, eicosapentaenoic acid; FA, fatty acid; LOX, lipoxygenases; LTB-4, leukotriene B-4; LxA4, lipoxin A4; LxB4, lipoxin B4; PGD2, prostaglandin D2; PGE2, prostaglandin E2; TxA2, thromboxane A2; 12/15 HETE, 12/15 hydroxyeicosatetraenoic acid.

Overall, there is a great variety of PUFAs metabolites which possess pro- and anti-apoptotic properties, play important roles in inflammation, modulate the immune responses and probably affect many yet unknown processes (Figure 1; Masoodi et al., 2015).

### PUFAs in inflammation

So far, the role of PUFAs in inflammation has been mainly explained by the action of AA. One of its most important metabolites are PGs, a heterogenous group of molecules produced by COX-1 and COX-2. Their common precursor is prostagandin H2 (PGH2) produced from AA and later metabolized into downstream metabolites (Félétou et al., 2011). The main pro-inflammatory one is prostaglandin E2 (PGE2), produced in large quantities by macrophages and neutrophils in response to inflammatory stimuli. PGE2 induces fever, increases vascular permeability and vasodilation, and intensifies pain and oedema mediated by other inflammatory factors, such as histamine and bradykinin. It also enhances its own synthesis and induces production of IL-6 in macrophages. Moreover, PGE2 also plays a role in inducing immune tolerance in the intestine, not mediated by IL-10 or T regulatory cells (Stenson, 2014). Interestingly, prostaglandin D2 (PGD2), displays a strong, purely anti-inflammatory effect (Stenson, 2014) acting through two types of prostaglandin D2 receptors (DP), DP1 and DP2, the latter being identified as a member of the chemokine receptor family (Félétou et al., 2011). Can also be dehydrated to produce prostagandin J2 (PGJ2), involved in the differentiation of adipocytes by activation of PPARγ nuclear receptor (Félétou et al., 2011).

Besides PGs, the main AA metabolites are thromboxane A2 (TxA2), leukotriene B4 (LTB4) and LXs. TxA2 mainly promotes platelet aggregation and vasoconstriction but is also reportedly involved in allergies, modulation of acquired immunity, atherogenesis, neovascularization, and metastasis of cancer cells (Félétou et al., 2011). LTB4, in turn, recruits neutrophils, promotes vascular leakage, and regulates epithelial barrier function (Cornejo-García et al., 2016), whereas LXs diminish the inflammatory process by limiting leukocyte infiltration (Maderna and Godson, 2009). This shows that n-6 derivatives can both exacerbate and resolve inflammation. In contrast, n-3 PUFAs are considered purely anti-inflammatory FAs, because they serve as substrates for PLA2 in lipid membranes. This causes reduction in AA-derived pro-inflammatory cytokines and suppresses the inflammatory process (Okada-Iwabu et al., 2013).

Nevertheless, PUFAs metabolites gather increasing recognition as prime drivers of inflammation and its resolution. For example, 12-lipooxygenase (12-LO) plays a role in atherosclerosis and adipose tissue inflammation in obesity (Masoodi et al., 2015). In a rat model of obesity, Chakrabarti et al. (2011) reported increased expression of 5- and 12-LO in visceral adipocytes, along with increased amounts of their products: 12- and 5- HETE, and LTB-4.

In addition to pro- and anti-inflammatory properties of PUFAs derivatives, they can also exhibit pro-resolution effects. Contrary to well-established beliefs, resolution of inflammation is also an active process rather than simple cessation of inflammatory stimuli (Shinohara and Serhan, 2016). It is mediated by specialized pro-resolving molecules (LXs, Rvs, protectins, and maresins) derived from n-3 PUFAs, mainly EPA and DHA (Serhan, 2014). All these molecules mitigate neutrophil influx, stimulate phagocytosis and enhance efferocytosis of cellular debris. Additionally, they also exert class-specific influence on inflammation processes; they counteract eicosanoids, chemokines, and cytokines, regulate specific micro RNAs and act in a receptor-specific manner on human neutrophils and macrophages. In line, administration of RvD1 and 17-hydroxy-DHA to obese diabetic mice not only improved insulin sensitivity but also reduced levels of inflammatory cytokines in WAT and decreased its infiltration by macrophages. Importantly, pro-resolving molecules stimulate heme oxygenase system to produce low concentrations of carbon oxide which acts protectively for tissues (Shinohara and Serhan, 2016).

Another example of signaling molecules involved in inflammatory pathways are endocannabinoids, a heterogeneous group of lipid mediators acting on classical and non-classical cannabinoid (CB) receptors. Two very important representatives of these ligands are N-arachidonoylethanolamine named anandamide (AEA), and 2-arachidonoylglycerol (2-AG), both derivatives of AA (Alhouayek and Muccioli, 2012). Both suppress mast cells activation, decrease the production of macrophages and pro-inflammatory cytokines, and modulate T-cell helper activity. Moreover, endocannabinoids play an important role in GI-related inflammation. In line, the activation of classical CB1 and CB2 receptors reduces inflammation-induced hypermotility and attenuates visceral hypersensitivity (Sanson et al., 2006). In colonic cell lines, endocannabinoids promote wound closure, thus becoming an attractive target for treating IBD-related lesions (Alhouayek and Muccioli, 2012).

Overall, PUFAs offer many ways to modulate inflammation. They have also been linked with IBD on an epidemiological level. Analysis of the European Prospective Investigation of Cancer (EPIC)-Norfolk cohort (*N* = 25639) found a positive association of dietary AA intake with ulcerative colitis (UC) incidence and a protective effect of oleic acid intake (de Silva et al., 2014).

## PUFAs in inflammatory bowel diseases

Inflammation is a biological response that occurs in many GI disorders and simultaneously underlies both “classical” diseases, such as non-alcoholic fatty liver disease (NAFLD) and non-specific IBD including UC and Crohn's disease (CD). NAFLD is characterized by a cryptic low-grade inflammation process affecting the liver, with concomitant features of metabolic syndrome: obesity, dyslipidemia, insulin resistance and hypertension (Xu et al., 2015; Yki-järvinen, 2015). NAFLD affects patient's lifespan by elevating the risk of cardiovascular disease (CVD) (Xu et al., 2015; Yki-järvinen, 2015). The symptoms of NAFLD are usually scarce and the treatment is mainly symptomatic—it includes losing weight, increase in physical activity and diet modifications (Yki-järvinen, 2015). n-3 PUFAs are considered a relevant supplementation in patients with hypertriglyceridemia and many studies suggest the important role of PUFAs in NAFLD pathophysiology (Gambino et al., 2016). However, there is no consensus from human trials for their systematic use as a drug treatment (Boyraz, 2015; Nakamoto et al., 2016). Nevertheless, this aspect is not the subject of this review; for detailed information please see (Boyraz, 2015; Yki-järvinen, 2015; Musso et al., 2016).

In contrast to NAFLD, the symptoms of UC and CD are very pronounced and significantly affect patient‘s quality of life (Sobczak et al., 2014). The most common symptoms are diarrhea, fever and fatigue, abdominal pain and cramping, intestinal bleeding, reduced appetite and unintended weight loss. Their core is a constant, low-grade inflammation with temporary exacerbations. Both UC and CD affect 37 adults and 5-11 children per 100 000 annually, leading to increased mortality (Ananthakrishnan, 2015; Ye et al., 2015). The pathogenesis of IBD is not completely defined, but recently the spotlight is on microbiota-stimulated immunoreactivity and adipose tissue hormonal dysregulation (Kostic et al., 2014; Schaubeck and Haller, 2015). What is more important, the currently available treatment includes 5-aminosalicylates, steroids, biological therapy and surgical procedures—all aggressive and with significant side effects (George et al., 2015; Khanna and Feagan, 2015; Meyer et al., 2015). Thus, it is crucial to seek new agents that could influence the clinical course of IBD and alleviate or prevent exacerbations. In this field, PUFAs are recognized as promising agents.

### Animal studies

The impact of PUFAs on colitis-associated inflammation has been extensively studied in animal models (Huang et al., 2016). To date, oral administration of dextran sulfate sodium (DSS) and intracolonic administration of 2,4,6-trinitrobenzenesulfonic acid (TNBS) dissolved in ethanol are two most widely used models to induce UC and CD, respectively (Scheiffele and Fuss, 2002; Chassaing et al., 2014; Sałaga et al., 2014a,c; Salaga et al., 2016). The main difference between these two models of colitis is that DSS directly damages gut epithelial cells of the basal crypts and disrupts the integrity of the mucosal barrier, whereas in TNBS model, ethanol breaks mucosal barrier and therefore allows TNBS to haptenize colonic proteins in order to stimulate the immune response.

One of the first targets in fighting inflammation may be conjugated LA (CLA) (Viladomiu et al., 2013). The mixture of LA isomers has been categorized by Food and Drug Administration (FDA) as “generally recognized as safe” (GRAS) and can be used as a supplement. In pig models of colitis, CLA was proved to downregulate inflammation by lowering serum levels of tumor necrosis factor alpha (TNF-α) and nuclear factor κB (NF-κB) while increasing levels of transforming growth factor β (TGF-β) and upregulating the expression of peroxisome proliferator-activated receptor γ (PPAR-γ) (Hontecillas et al., 2002). These findings were confirmed in DSS- and CD4-induced colitis in mice (Bassaganya-Riera et al., 2004).

Another idea of curbing colonic inflammation is to utilize n-3 PUFAs. This is based on the observation that transgenic mice with inborn ability to convert n-6 PUFA to n-3 PUFA were protected from experimental colitis. Furthermore, in a rat model of TNBS-induced colitis, diet supplemented with ALA decreased the number of lesions, normalized colon induced NO synthase (iNOS) and COX-2 expression and lowered levels of TNF-α and leukotriene B-4 (LTB-4) to baseline (Hassan et al., 2010). However, it had no effect on PGE2 level and, more importantly, it did not lower the disease inflammation score (Hassan et al., 2010). In TNBS model, ALA supplementation was also proved to decrease the levels of intercellular adhesion molecule 1 (ICAM-1), vascular cell adhesion protein 1 (VCAM-1) and vascular endothelial growth factor receptor 2 (VEGFR-2), thus suppressing the angiogenic component of inflammation (Ibrahim et al., 2012).

Other studies concentrate on n-6 to n-3 PUFAs ratio rather than one particular FA. An increase in western high-fat diet consumption worldwide results in dramatic changes in this ratio of FAs from 2:1 in the past, to 10:1 nowadays (Tyagi et al., 2012). In DSS-induced colitis replacement of dietary LA with ALA, and therefore decreasing LA/ALA ratio to 2:1, reduced the severity of colitis and significantly mitigated inflammation, when measured indirectly as shortening of the colon (Tyagi et al., 2012). This change was probably caused by decreased neutrophil infiltration into the colonic tissue, probed by the decrease in myeloperoxidase (MPO) and colonic alkaline phosphatase activity. Supplementation with ALA also prevented the rise in TNF-α and IL-1β levels, when compared to the control group. Moreover, this diet also partially decreased plasma NO and completely prevented the rise in radical stress in colonic tissue. A reduction in histological score of inflammation was also observed (Tyagi et al., 2012).

Another way to modulate inflammation is to use specific PUFAs metabolites rather than primary FAs. Given the fact that adipose tissue inflammation plays an important role in CD (Michalak et al., 2016), these metabolites may be actively used to suppress existing inflammation or prolong remission in those patients. EPA- and DHA-derived specialized pro-resolving mediators are prime candidates for animal and clinical trials.

A double role of PGE2 in inflammation has also been demonstrated in animals. In DSS-induced colitis PGE2 enhanced proliferation and epithelial healing, whereas in TNBS-induced colitis it promoted Th17 differentiation pathway in lymphocytes and thus exacerbated inflammation (Stenson, 2014). These seemingly inconsistent actions of PGE2 may result from profound biological differences in DSS- and TNBS-induced colitis.

Another group of PUFAs derivatives tested in animal models of IBD are agents targeting the endocannabinoid system. Firstly, it has been demonstrated that CB1- and CB2-knockout mice show increased susceptibility to DSS-induced colitis (Alhouayek and Muccioli, 2012). In line, administration of cannabinoids or inhibitors of cannabinoid degradation decreased intestinal motility, peristalsis and colonic propulsion in rodents in a CB1-dependant manner (Alhouayek and Muccioli, 2012). Sałaga et al. (2014c) thoroughly tested fatty acid amide hydrolase (FAAH) inhibitor in TNBS- and DSS-induced colitis. In the TNBS model, orally administered FAAH inhibitor exerted potent anti-inflammatory effect which improved total histological score and decreased tissue MPO activity. Such changes were not observed in the DSS model, probably due to different mechanisms involved in the inflammatory process (Sałaga et al., 2014b,c).

When assessing possible use of PUFAs in therapy, it may be worth to also consider alternative ways of delivery. One of the newest methods includes the encapsulation of n-3 PUFA in liposomes. Notably, by adding radioactive, fluorescent or superparamagnetic agents it is possible to track the drug distribution in the organism and better study its local actions (Calle et al., 2015). Liposomes were already tested in DSS-induced colitis medium, which serves to deliver glucocorticosteroids; unfortunately, this therapy led to worsening of fecal blood loss (Crielaard et al., 2011). However, when imbued with n-3 PUFA, liposomes demonstrated a clear anti-inflammatory effect in DSS-induced colitis in mice and exerted important anti-tumoral effects against glioma. Imaging techniques confirmed the presence of magnetoliposomes in inflamed regions, which opens up many possibilities for targeted, image-guided delivery of medical agents, which may find application in IBD therapy (Calle et al., 2015).

The last study that needs to be mentioned in this chapter may be considered as a bridge toward clinical trials. Meister and Ghosh (2005) incubated biopsies from IBD patients with fish oil and found reduced inflammation manifested by a rise in IL-1a/IL-1b ratio in tissues obtained from UC, but not from CD patients. These outcomes indicate differences in a diet that should be taken into consideration while composing nutritional therapy for UC or CD patients.

### Clinical application

Despite promising results in animal studies, translation of PUFA-based interventions into humans is difficult. One of the attempts was to utilize PUFAs against chronic low-grade inflammation in obesity. It has been demonstrated that obesogenic diet concomitant with EPA/DHA supplementation resulted in a more favorable metabolic profile and normalized the levels of endogenous Rvs and protectins, attenuated adipose tissue inflammation and improved insulin sensitivity (Minihane et al., 2015). Another study proved that high dose of long-chain n-3 PUFA, delivered to severely obese non-diabetic patients, increased secretion of anti-inflammatory eicosanoids and decreased expression of inflammatory genes in subcutaneous adipose tissue Finally, a systematic review by Rangel-Huerta et al. (2012) reported that n-3 PUFAs supplementation affects inflammatory biomarkers in cardiovascular disease and chronic and acute conditions. However, its effects varied in disease-specific manner.

Polyunsaturated fatty acids (PUFAs) have also been evaluated as a factor involved in the course of IBD. Total dietary intake of PUFAs has been shown to positively correlate with the risk of UC, although with marginal significance and without distinguishing between n-3 and n-6 PUFA (Hart et al., 2008). A more detailed big cohort study demonstrated that total dietary n-3 PUFAs, particularly EPA and DHA, protected from UC development in patients older than 45 years (John et al., 2010). Finally, a large prospective cohort study in women also proved the association between higher intake of dietary long chain n-3 PUFAs and a reduced risk of UC (Ananthakrishnan et al., 2014). Noteworthy, the latest case-control trial reported that dietary intake of fats, especially PUFAs, is associated with increased risk of UC; however, no positive association was seen specifically for n-3 PUFAs intake (Rashvand et al., 2015). Altogether, the above-mentioned studies suggest that n-6 PUFAs facilitate the development of UC while n-3 PUFAs possibly prevent it. This leads to assumption that n-3 PUFAs could be utilized either in inducing or prolonging remission in IBD. Important clinical trials together with summary meta-analysis have been listed in Table 1.

**Table 1:** **Clinical trials investigating the use of n-3 PUFAs in IBD**.

**Condition**	**Study design**	**Participants**	**Intervention**	**Outcomes**	**References**
IBD-related joint pain	RCT (10 d. treatment period)	17 (9 CD, 10 UC)	Seal oil (*n* = 10) vs. soy oil (*n* = 9) 10 ml 3x daily, administered duodenally	Reduced bodily pain	Bjørkkjær et al., 2006
Active CD	Open label (12 weeks of treatment)	13	CLA 6 g/d	Drop in CD activity index, increase in quality of life	Bassaganya-Riera et al., 2004
CD (patients with currently or recently raised inflammatory markers)	RCT (24 weeks of treatment)	61	EPA and DHA (+ antioxidants) 2.7 g/d for (*n* = 31), Placebo (*n* = 30)	reduction in IFNγ production by mitogen-stimulated PBMC	Trebble et al., 2004
Pediatric patients with CD in remission	Double-blind RCT (12 months of treatment)	38	5 ASA (50 mg/kg/d) + EPA + DHA (3 g/d) vs. 5-ASA (50 mg/kg/d) + olive oil (3 g/d)	Lower relapse rate after 1 year	Romano et al., 2005
Active CD	Double-blind RTC (9 weeks of treatment)	31	Nutritional treatment with an isocaloric diet Impact Powder (3 g omega-3 FA, 11.4 g L-Arginine, and 1.2 g RNA per day) Control formula: Nutritional supplement with 7.8 g linoleic acid per day (all patients received systemic steroid therapy)	Significant decrease in CDAI and C-reactive protein in both groups (no difference)	Nielsen et al., 2005
Active UC	Double-blind RTC (6 months of treatment)	121 (86 completed the protocol)	Nutritional supplement with <2.5 g EPA and <1.0 g DHA per day vs. supplement based on sucrose alone (Steroids and 5-ASA allowed, their doses adjusted to clinical response)	Similar clinical improvement, but faster reduction in steroid dose in active group	Seinder et al., 2005
Newly-diagnosed pediatric patients with CD	Double-blind RTC (6 weeks of treatment)	41	Polymeric diet with 1.5% of energy as ALA and 3% as LA vs. elemental diet with 0.4% of energy ALA and 5.4% as LA (No other active therapy for CD allowed)	Similar remission rate between the groups	Grogan et al., 2012
CD patients immediately after reaching remission	Double-blind RTC (58 weeks of treatment)	375	Enteric coated capsules (2-2.4 g EPA/day and 0.6–1 g DHA/day) vs. placebo capsules	47.8% relapse rate vs. 48.8% in placebo	Feagan et al., 2008
CD patients in remission	Double-blind RTC (52 weeks of treatment)	363	Enteric coated capsules (2–2.4 g EPA/day and 0.6–1 g DHA/day) vs. placebo capsules	31.6% relapse rate vs. 35.7% in placebo group	Feagan et al., 2008
CD patients in remission	Metaanalysis of RCT (at least 6 months of treatment)	1039	Fish oil or n-3 PUFA Predefined doses	Risk of relapse - RR 0.77, 95% CI 0.61 to 0.98	Turner et al., 2011 Lev-Tzion et al., 2014
					Cabré et al., 2012

*ASA, amino salicylic acid; CD, Crohn disease; CLA, conjugated linoleic acid; DHA, docosahexaenoic acid; EPA, eicosapentaenoic acid; IBD, inflammatory bowel disease; IFNγ, interferon-gamma; PUFA, polyunsaturated fatty acid; RCT, randomized controlled trial*.

In patients with an active form of IBD, administration of duodenal seal oil significantly reduced disease activity index, normalized n-3/n-6 PUFAs ratio and alleviated pain, especially joint-related pain (Arslan et al., 2002; Bjørkkjær et al., 2006). Worth mentioning, in patients with mild to moderate CD, the supplementation with CLA suppressed the ability of T-cells to produce TNF-α, IFN-γ, and IL-17, which decreased disease activity and improved the quality of life of patients (Viladomiu et al., 2013).

When joined with antioxidants, n-3 PUFAs supplementation altered the composition and function of peripheral blood mononuclear cells (PBMCs). Consequently, when stimulated with mitogens, PBMCs produced less IFN-γ, but when stimulated by lipopolysaccharide generated less PGE2 (Trebble et al., 2004).

Possible clinical application of PUFAs has been assessed by a few systematic reviews and meta-analyses. The study by Turner et al. (2011) evaluated the effectiveness of n-3 PUFAs in maintaining remission in patients with IBD. They found moderate but significant beneficial effect of omega FA supplementation in patients with CD (relative risk 0.77; 95% confidence interval 0.61–0.98) (Turner et al., 2011). However, conclusions were drawn from only 6 trials comprising 1039 patients altogether, with probably high heterogeneity and publication bias. Analysis of 3 trials on supplementation in 138 patients with UC did not report any significant effect. The same or similar analysis was probably republished in 2014 concluding that n-3 PUFAs may be ineffective for maintenance of remission in CD (Lev-Tzion et al., 2014).

Another systematic review published in 2012 confirmed ineffectiveness of n-3 PUFA supplementation in remission phase of UC or CD. Nonetheless, the supplementation showed many beneficial effects in patients with active phase of UC. Unfortunately, due to high variability of studies and high number of parameters assessed, these results could not be unified (Cabré et al., 2012).

The reason for the shortcomings of the intervention trials may be related to different doses applied in each study and complex interfering metabolic reactions that PUFAs undergo before exerting their biological effects. The key to unlock its power is to utilize alternative, effective ways of drug delivery, such as liposomes. This would allow to evaluate the effects of EPA or DHA action in specific inflammatory environment and thus open up endless possibilities of using particular n-3 PUFA metabolites to achieve more specific effects.

n-3 PUFAs supplementation may also be an attractive option in pediatric patients. In children with CD receiving mesalazine, supplementation with triglyceride form of n-3 PUFAs in gastro-resistant capsules, containing 400 mg/g of EPA and 200 mg/g of DHA, significantly lowered the relapse rate within 1-year observation with respect to patients supplemented with olive oil placebo capsules (Romano et al., 2005).

Currently, there is no strong evidence that oral n-3 PUFAs supplementation significantly changes the course of either CD or UC, enabling clinicians to drop steroid or 5-ASA therapy. However, n-3 PUFAs may be valuable additions to standard treatment, but more uniformly-devised studies are warranted.

## PUFAs in irritable bowel syndrome

Dietary fat also plays an important role in FGIDs (Feinle-Bisset and Azpiroz, 2013). This heterogenous group is generally defined as conditions with a variable combination of chronic or recurrent GI symptoms. Irritable bowel syndrome (IBS) is the most prevalent FGID noted in the general population, which constitutes 25–50% of gastroenterology outpatients' workload (Wilson et al., 2004). According to the Rome IV criteria, a symptom-based classification system, the clinical symptoms of IBS include abdominal pain, bloating, stool irregularities with concomitant psychiatric and somatic comorbidities (Drossman et al., 2010; Palsson et al., 2016). Depending on the pattern of symptoms, IBS may be classified as diarrhea-predominant IBS (IBS-D), constipation-predominant IBS (IBS-C) or alternating IBS (IBS-A). Among others, one of the putative theories that underlie IBS development is the imbalance within the brain-gut axis, made up of the enteric nervous system (ENS), central nervous system (CNS), and the hypothalamo-pituitary-adrenal axis (Fichna and Storr, 2012). Such imbalance may be triggered by early life stress, which predisposes to develop stress related disorders, including IBS later in life. A number of studies reported low-grade inflammation as another mechanism implicated in IBS, in which higher infiltration of cytokines e.g., IL-4, IL-6, or TNF-α, pro-inflammatory mediators and mast cells in the colonic mucosa impairs the tight junctions complexity and thus exacerbates IBS symptoms (Camilleri et al., 2012).

The management of IBS requires multimodal approach, including pharmacological, psychological, as well as complementary and alternative medicines. Approved and investigated therapeutics are summarized in Table 2.

**Table 2:** **Currently available and emerging pharmacological treatment options for IBS-C and IBS-D (Sweetser et al., 2009; Chey et al., 2011; Mozaffari et al., 2013; Mosinska et al., 2015, 2016; Shailubhai et al., 2015)**.

**Drug class**	**Generic name**	**Mechanism of action**	**Indication**	**ClinicalTrials.gov Identifier**
BA modulators	Elobixibat	IBAT inhibitor	IBS-C	NCT01833065
	NaCDC	BA analog	IBS-C	NCT00912301
Prokinetics	Itopride	ACh esterase inhibitor; D2 receptor antagonist	IBS-C	NCT01027260
	Relenopride	5-HT4 partial agonist	IBS-C	NCT02082457
	Mosapride	5-HT4 agonist	IBS-C	NCT01505777
	Tegaserod			approved
	Velusetrag			NCT00391820
	Renzapride	5HT4 agonist and 5HT2b and 5HT3 antagonist	IBS-C	NCT00268879
	Pumosetrag	5-HT3 agonist	IBS-C	not available
	Alosetron	5-HT3 antagonist	IBS-D	NCT00067457
	Ondansetron			not available
	Ramosetron			approved in Japan, Korea and Thailand
PAMORA	Loperamide	μ-opioid receptor agonist	IBS-C	approved
	Eluxadoline	μ-opioid receptor agonist, δ-opioid receptor antagonist	IBS-D	approved
Secretagogue	Dolcantide	GC-C agonist	IBS-C	not available
	Linaclotide			approved in USA, EU
	Plecanatide			NCT01722318
	Lubiprostone	CIC2 activator	IBS-C	approved in USA
**AGENTS WITH OTHER MECHANISMS OF ACTION**				
	TC6499	Alpha_3_beta_4_ NNRs modulator	IBS-C	NCT01149200
	Taranabant	CB1 receptor agonist	IBS-C	not available
	Crofelemer	Chloride channel inhibitor	IBS-D	NCT00461526
	Pexacerfont	CRF receptor antagonist	IBS-D	NCT00399438
	ROSE-010	GLP-1 Analog	IBS-C	NCT01056107
	TU-100	Kampo medicine	IBS-C	NCT01890837
	Asimadoline	κ- opioid agonist	IBS-D	NCT01100684
	Ibodutant	NK2 antagonist	IBS-D	NCT01303224
	NHE3 inhibitor	Tenapanor	IBS-C	NCT02727751

Currently, dietary interventions are gaining much attention (Böhn et al., 2013; Cuomo et al., 2014). In line, a diet excluding foods high in short-chain carbohydrates termed FODMAPs (Fermentable Oligo-, Di- and Monosaccharides and Polyols) has proven its effectiveness in alleviating IBS symptoms. More recent evidence also indicates the usefulness of dietary lipids (Caldarella et al., 2005; Solakivi et al., 2011; Halmos et al., 2014).

### Animal studies

Visceral hypersensitivity can be mimicked in animal model of IBS. Rats, when separated as neonates from their mother and exposed in later life to acute stress (e.g., in the water avoidance test) display hypersensitivity to mechanical colorectal distension that can be measured electromyographically, and manifest increase in visceral pain. In these animals, elevated levels of pro-inflammatory PUFAs were reported (Clarke et al., 2009). However, in maternally separated rats, dietary supplementation with fish oil reduces neither the response to colon distension nor the level of pro-inflammatory cytokines in colonic tissue (van Diest et al., 2015). Another study combined the supplementation with CLA and probiotics (*B.breve* DPC6330) (Barrett et al., 2012). Groups of 15 rats were either separated from their mothers or not, and fed with probiotic, probiotic together with LA and ALA, or placebo. The supplementation provided significant changes in lipid composition in the rats' serum, colonic tissue and prefrontal cortex, but failed to clearly change the colonic hypersensitivity measured by colon distension (Barrett et al., 2012). Of note, the use of human intestine bacteria (*B. breve, strain* DPC6330) affected the FA metabolism in maternally separated rats to a greater extent than in non-separated animals. The study thus supports the hypothesis that changes in the gut microbiota alter host lipid (e.g., palmitoleic acid, DHA or propionate) composition, and are responsible for the occurrence of IBS symptoms.

### Clinical application

Polyunsaturated fatty acids (PUFAs) can modulate mast cells activity and further mitigate visceral hypersensitivity, a prime feature of IBS. This leads to the question whether supplementing PUFAs could help change the clinical course of IBS and improve patients quality of life. The study by Clarke et al. (2010) reported significant increase in the level of AA and its metabolites in serum samples from IBS patients, compared with healthy controls. An elevated concentration of LTB4 was found across all IBS subtypes, whereas PGE2 showed type-specific elevation associated solely with IBS-D. It should be noted that PGE2 is able to cross the blood brain barrier, which links FA metabolism and inflammation to brain-gut axis, and therefore may be implicated in the course of IBS (Clarke et al., 2010). There was no correlation between the plasma level of AA and symptom severity. Surprisingly, the same study also found increased concentrations of n-3 PUFAs in serum of IBS patients, however the underlying cause of this augmentation remains unexplained (Clarke et al., 2010).

Polyunsaturated fatty acids (PUFAs) metabolites can bind TRP ion channels, such as transient receptor potential vanilloid 1 (TRPV1), TRPV4, and TRP subfamily A receptor 1 (TRPA1), and signal to sensory neurons to evoke hypersensitivity symptoms seen in IBS. The levels of TRP agonists differ between colon biopsies from IBS patients and healthy individuals. Cenac et al. (2015) demonstrated an increased concentration of a TRPV4 agonist, 5,6-epoxyeicosatrienoic acid (5,6-EET), but not TRPA1 and TRPV4 agonists, in colonic biopsies from IBS-D patients, when compared with control group. This augmentation in 5,6-EET was positively correlated to pain severity and bloating. Biopsies from both IBS-C and IBS-D patients showed elevated levels of PGE-2 and decreased concentration of a TRPV1 agonist. Modulating TRP activity by PUFAs or PUFAs-based ligands may potentiate the production of TRPV4 and its agonist and thus modulate visceral sensation in IBS.

The same study proved that intracolonic administration of supernatants from IBS-D patients' colonic biopsies to mice led to development of hypersensitivity (manifesting as allodynia and hyperalgesia) and increased 5,6-EET, PGE-2 and 15-HETE levels in mouse colonic tissues (Cenac et al., 2015). These changes were prevented by using TRPV4 knockouts or pretreating mice with TRPV4 inhibitors. Finally, the study proved that 5,6-EET stimulates colonic neurons by acting on their TRPV4; the same effect was achieved later with bioptic supernatants from IBS-D patients and hypersensitive mice. The study highlights the potential of PUFA-based therapies in targeting TRPV4 channels.

Currently, there are no clinical trials assessing effects of PUFAs supplementation on clinical course of IBS. Such studies are warranted given the recently-revealed inflammatory factor in IBS.

Apart from supplementation, PUFAs may also serve as base for devising new biologically active agents. Of note, lubiprostone, a PGE1 derivative, has been registered in the treatment of IBS-C and chronic idiopathic constipation (CIC). It activates type 2 chloride channels in apical membrane of intestinal epithelium and thus increases fluid secretion in the GI tract (Li et al., 2016). Lubiprostone also promotes repair of epithelial barrier (Cuppoletti et al., 2012). Most recent metaanalysis by Li et al. (2016) confirmed the effectiveness and safety of lubiprostone in diminishing the severity of constipation and improving stool consistency, degree of abdominal pain and discomfort. Compared to alternative therapies, patients on lubiprostone have improved health-related quality of life, symptom control, reduced symptoms severity and are more satisfied with their therapy (Solem et al., 2016).

## PUFAs in colorectal cancer

Colorectal cancer (CRC) is the third most common cancer both in men and women, and second in cancer-related deaths (Yan et al., 2016). The risk of CRC is strongly affected by environmental modifiable factors, such as exercise or dietary habits (Teixeira et al., 2014). Fatty acid intake with focus on n-3 PUFAs is one of the main candidate factors affecting CRC incidence and clinical course. It is generally accepted that n-3 PUFAs are associated with protection from CRC, and n-6 PUFAs with its promotion. However, there is a limited number of studies undermining this paradigm and suggesting a more complicated role of PUFAs (Zhang et al., 2015b).

### Primary prevention

The primary prevention studies yield mixed results. A large 2 × 2 factorial study showed no association between the therapy with insulin glargine or supplementation with n-3 PUFAs and CRC incidence rate (Bordeleau et al., 2014). Similarly, metaanalysis by Shen et al. (2012) did not reveal any significant impact of n-3 PUFAs intake on CRC risk in general. Importantly, the subgroup analysis revealed a significantly reduced risk of CRC among men but this aspect needs further investigation (Shen et al., 2012). In contrast, metaanalyses of prospective cohort studies that investigated the impact of fish consumption or n-3 FAs on CRC prevalence and mortality, provided evidence that fish intake (at least once per week) inhibits CRC carcinogenesis and can lower risk of CRC for about 4% (Park et al., 2013).

On the other hand, PUFAs can still be used as adjuvant agents in therapy or as prevention in high-risk groups, for example in patients with familial adenomatous polyposis, or as a secondary prevention.

### Colon cancer cell lines

In human CRC cell lines, n-3 PUFAs modulate both p53-dependent and independent pathways, inhibit COX2 pathway, suppress NF-κβ and downregulate Bcl-2 expression suggesting its anti-proliferative and pro-apoptotic properties (Collett et al., 2001; Sala-Vila et al., 2010; Eltweri et al., 2016). The action of PUFAs on CRC cells is mediated by mitochondrial-dependent pathways and varies with a degree of cell differentiation. Zhang et al. (2015a) demonstrated that both n-3 and n-6 PUFAs induced apoptosis in LoVo and RKO CRC cell lines when applied at concentrations above 120 μM (tested at 0-200 μM). The pro-apoptotic effect of PUFAs is greater in semi-differentiated RKO line compared with undifferentiated LoVo cells. Furthermore, DHA and to lesser extent EPA, enhanced the effects of radiotherapy in two human CRC cell lines (Cai et al., 2014).

Another study by De Carlo et al. (2013) investigated cell-specific action of n-3 PUFAs on differentiated tumor cells and cancer stem like cells (CSLC). The treatment decreased the expression of CD133 in CD133+ colon CSLCs, what suggests a differentiation-stimulating effect (De Carlo et al., 2013). Moreover, EPA also sensitized CRC cells to chemotherapeutics, 5-fluorouracyl, and oxaliplatin. An increased sensitivity to 5-fluorouracyl was found in CSLCs (De Carlo et al., 2013). Another study demonstrated that EPA coupled with these two cytostatics inhibits cell growth, colonosphere formation, sphere-forming frequency and increases the sphere disintegration in otherwise resistant cells (Vasudevan et al., 2014). Similar synergistic interaction of n-3 PUFAs with chemotherapeutic agents was also supported by other authors; for more information, please see: (Jordan and Stein, 2003; Cai et al., 2014; Eltweri et al., 2016). Currently, 5-fluorouracyl and oxaliplatin are first line of treatment in CRC and EPA might be considered a valuable addition. However, clinical trials are needed to determine if the sensitizing effect of EPA is present and clinically relevant *in vivo*.

Additionally, PUFAs also modulate the inflammatory environment in CRC patients. The serum level of n-6 C20:4 PUFAs show positive association with IL-6 and inverse with levels of metalloproteinase-7. In line, there is a positive correlation between C22:5 n-3 PUFAs and IFN-γ, inverse association between C20:5 n-3 PUFA and metalloproteinase-2 and C22:6 n-3 PUFA and levels of IL-8 and metalloproteinase-9 (Jia et al., 2016). This suggests that nutritional intervention may influence the inflammatory signaling and clinical course of CRC.

### Animal models

The *in vivo* results are consistent with *in vitro* studies. In mice and rats, EPA and DHA supplementation reduces cell proliferation and promotes apoptosis (Gutt et al., 2007). This inhibits liver metastasis and leads to decrease in the tumor volume. Importantly, hybrid liposomes (HLs) including PUFAs have demonstrated an inhibitory effect on the growth of tumor cells both *in vitro* and *in vivo* (Ichihara et al., 2014). Tanaka et al. (2008) demonstrated that HLs enriched with n-3, n-6, and n-9 PUFAs inhibit the growth of tumor cells, including colon tumor (WiDr) cells *in vitro*. The highest inhibitory effect was seen in HLs enriched with n-3 PUFA DHA; this action could be attained mostly by increased peroxidation and further necrosis of the cell. However, the effect of DHA-HLs was not totally prevented by addition of antioxidant so their mechanism of action is likely to be more complicated, especially that DHA also demonstrated a pro-apoptotic effect in WiDr colon tumor cells (Tanaka et al., 2008).

More recently, Ichihara et al. reported therapeutic effects of cationic HLs on the hepatic metastases of CRC along with apoptosis in mice. Female mice with severe combined immunodeficiency were injected intrasplenically with HCT116 cells (5.0 × 10^6^ cells). They were then randomized for intravenous administration of liposomes, containing either L-α-dimyristoylphosphatidylcholine (DMPC) (136 mg/kg/d) or 50% molar DHA (65.7 mg/kg/d) and 50% molar DMPC. After 14 days of therapy, a significant improvement in median survival time and induction of apoptosis in liver metastases were observed (Ichihara et al., 2014).

It is possible that liposome-delivered PUFAs may exert more potent effects on cancer cells than its oral administration; however, this needs further testing and clinical trials. Currently, only orally-supplemented PUFAs are available, but their various forms differ in the efficacy of their absorption from the intestine and incorporation into cells. The EPA in the free fatty acid (EPA-FFA) form is absorbed better from small intestine than the ethyl ester or triglyceride form (Lawson and Hughes, 1988). *In vitro*, EPA-FFA induces apoptosis of HCA-7 human CRC cells by decreasing PGE2 and increasing PGE3 synthesis, what affects their subsequent action on EP4 receptor (Hawcroft et al., 2010). Alone, PGE3 acts as a partial receptor agonist but in the presence of its natural ligand, PGE2, it competes for the receptor and acts as an antagonist. Importantly, HT-29 human CRC cells with EP4 overexpression (HT-29-EP4) do not produce detectable amounts of PGE2 or PGE3 and exhibit no reaction to treatment with EPA-FFA (Hawcroft et al., 2010).

Possibly, an even better form of delivering PUFAs are monoacylglycerols. Morin et al. (2013) showed that this form can be more easily absorbed than FFA. *In vitro*, monoacylglycerol used to deliver docosapentaenoic acid (DPA), an intermediate product between EPA and DHA, to CRC cell lines showed more potent anti-proliferative and pro-apoptotic effect than either EPA or DHA. The action of DPA was also confirmed in a mouse xenograft model of CRC (Morin et al., 2013).

Gounaris et al. (2015) proposed another way to target PUFA metabolism in CRC. In a mouse model of polyposis, the administration of Zileuton (5-LO inhibitor registered for threating asthma) resulted in markedly lowered systemic and local lesion inflammation and led to a decrease in polyp burden (Gounaris et al., 2015). This warrants clinical studies of agents influencing PUFA metabolism in patients with high risk of CRC.

### Clinical application

In humans, n-3 PUFAs have been tested as adjuvants to standard therapy, components of nutritional treatment and supplements for prevention in high-risk groups. Important clinical studies from each field has been summed up in Table 3.

**Table 3:** **Recent clinical reports on n-3 PUFA as primary prevention supplements, prospective therapeutics, adjuvants to chemotherapy, nutritional treatment, or prevention in high-risk groups**.

**n-3 PUFA in CRC**					
**Condition**	**Study design**	**Participants**	**Intervention**	**Outcomes**	**References**
**PRIMARY PREVENTION**					
Patients with diabetes, impaired glucose tolerance or impaired fasting glycemia	2 × 2 RCT (median follow-up 6.2 years)	12,536	Insulin: individual regimen, n-3 PUFA: 900 mg ethyl esters daily	No effect of n-3 PUFA intervention on CRC occurence	Bordeleau et al., 2014
**ADJUVANT TO CHEMOTHERAPY**					
Patients with CRC in chemotherapy	RCT (9 weeks of treatment)	11	2 g of fish oil/daily (600 mg/day EPA + DHA) vs. no supplementation	Reduced CRP/albumin ratio	Mocellin et al., 2013
**NUTRITIONAL TREATMENT**					
Patients with solid tumors	RCT (7 days of treatment)	38	400 ml of medical food, high in protein and leucine, enriched with fish oil and oligosaccharides vs. iso-caloric/iso-nitrogenous product	Reduced PGE2 levels	Faber et al., 2013
Patients referred for CRC elective surgery	Double-blind RCT (7 days of treatment)	138	Oral nutritional supplement (2 g EPA/day, 1 g DHA/day) vs. standard supplement	Increase in LTB5 and 5-HEPE production, decrease in LTB4 production by stimulated neutrophils, no effect on post-operative complications	Sorensen et al., 2014
**CLINICAL TRIAL IN METASTATIC CRC**					
Patients undergoing liver resection for CRC liver metastases	Double-blind RCT (median 30 days of treatment)	88	EPA-FFA 2 g daily vs. placebo	No difference in Ki-67 proliferative index, better overall survival	Cockbain et al., 2014
**PREVENTION IN HIGH-TISK GROUPS**					
Patients with FAP after colectomy	RCT (6 months of treatment)	55	EPA-FFA 500 mg twice daily vs. placebo	Reduction in polyp sizes, numbers and global polyp burden	West et al., 2010

*5-HEPE, 5-hydroxy-eicosapentaenoic acid; ASA, amino salicylic acid; CD, Crohn disease; CLA, conjugated linoleic acid; CRP, C-reactive protein; DHA, docosahexaenoic acid; EPA, eicosapentaenoic acid; FAP, familial adenomatous polyposis; IBD, inflammatory bowel disease; IFNγ, interferon-gamma; LTB5, leukotriene B-5; PGE2, prostaglandin E2; PUFA, polyunsaturated fatty acid; RCT, randomized controlled trial*.

When used as an addition to the treatment protocol, oral n-3 PUFAs supplementation (600 mg/day EPA + DHA) reduced inflammation in patients receiving chemotherapy (Mocellin et al., 2013). The results from a metaanalysis by Mocellin et al. (2015) supported n-3 PUFA supplementation as a way to diminish the inflammatory state in CRC patients, either by decreasing levels of IL-6 or CRP/albumin ratio. Moreover, Silva et al. (2012) showed that fish oil supplementation (2 g of fish oil, 600 mg/day EPA + DHA) prevented weight loss in patients undergoing chemotherapy, which may influence their quality of life and overall wellbeing. Additionally, another study by Faber et al. (2013) proved that supplementation of EPA (600 mg/day) with DHA (300 mg/day) in medical food to patients receiving radiotherapy effectively lowers serum levels of pro-inflammatory mediator PGE2.

In perioperative conditions, EPA (2 g/day) and DHA (1 g/day) in oral nutritional supplementation also showed anti-inflammatory effects by inducing the conversion of LTB5 to LTB4 in stimulated neutrophils (Sorensen et al., 2014). Nevertheless, the rate of surgical complications was similar between study and control group.

In phase II clinical trials, Cockbain et al. (2014) reported a direct effect of EPA-FFA intake on CRC cells. Patients undergoing liver resection for CRC liver metastases were supplemented with EPA-FFA (2 g/day) or placebo but this supplementation did not change the Ki-67, a proliferation marker index, in CRC. Nonetheless, the therapy was safe, well-tolerated, and reduced tumor vascularization and improved overall survival rate.

### PUFAs as possible prevention in high-risk groups

Age and sedentary lifestyle are established risk factors for developing CRC (Bonnington and Rutter, 2016). There are also hereditary conditions, such as familial adenomatous polyposis (FAP) which are associated with high number of possibly-malignant polyps (Brosens et al., 2015). In general population, detection of pre-cancerous polyps in colonoscopy is a risk factor for the development of CRC later in life (Bonnington and Rutter, 2016). All these patients are possible target groups for intervention with PUFAs. In patients with FAP, a 6-month-long supplementation with 2 g of EPA-FFA daily reduced the number of polyps by 22.4% and their size (diameter) by 29.8%. It also prevented the rise in global polyp burden, when compared with the non-supplemented group (+0.09 vs. −0.34, difference 0.42, statistically significant) (West et al., 2010). Recently, a multicenter double-blinded, placebo-controlled trial was conducted on patients with colonoscopy-detected polyps or aberrant crypti focci (ACF), supplemented with EPA 900 mg or placebo thrice daily for 1 month (Higurashi et al., 2012). The endpoints included formation of ACF, cell-proliferative and cell-apoptotic activity in colorectal polyps and normal mucosa. No results have been published so far; however, the trial has been registered in UMIN-CTR Search Clinical Trials as UMIN000008172.

Currently, there are also two randomized clinical trials evaluating a possible use of PUFA in patients at increased risk of CRC. The former investigates the effect of EPA and aspirin in a 2 × 2 factorial randomized controlled trial on colorectal adenoma (Hull et al., 2013). The target group includes patients at increased risk (55–74 y.o., >5 adenomas or >3 adenomas with at list one >10 mm diam. found at the first complete screening colonoscopy). The endpoints of the 12-month supplementation with EPA include, among others, the number of patients with one or more adenomas at surveillance colonoscopy, the number of advanced adenomas and the number of patients reclassified into intermediate risk (Current Control Trials ISRCTN05926847). The second clinical trial is conducted by Harvey Murff from Vanderbilt University (ClinicalTrials.gov Identifier: NCT01661764). It aims to recruit 150 participants with recently identified adenomatous polyps and conduct a 6-month double blind 3 × 2 factorial randomized controlled trial. The study will assess effects of fish oil supplementation in patients with three different polymorphisms in fatty acid desaturase 1 (FAD1) gene. FAD1 converts LA to AA and its activity determines the levels of AA in tissues. In individuals with inherently lower activity of FAD1, AA levels are substantially lower. The trial will evaluate the efficacy of fish oil supplements on rectal epithelial cell proliferation indexes (measured by Ki-67 labeling) and markers of rectal crypt apoptosis. Moreover, it will provide an insight whether the supplementation is more beneficial in patients with specified genotype. The completion date of this study is due December 2016. Our search also revealed other trials investigating the effect of PUFAs supplementation on clinical course of CRC and IBD; they are summarized in Table 4.

**Table 4:** **Summary of ongoing and recently completed clinical trials**.

**Status; ClinicalTrials.gov Identifier**	**Title**	**Study design**	**Indication**	**Diet and intervention**	**Primary endpoint**
Recruiting; NCT02534389	Fish oil supplement combined with neoadjuvant chemoradiation for locally advanced rectal cancer	I, R, OP	Rectal neoplasm	Omega-3 fish oil, 2.4 g of EPA + DHA vs. no intervention	Effects of daily consumption of 2.4 g of EPA + DHA for adults with rectal adenocarcinoma in neoadjuvant chemoradiation treatment on Glasgow Prognostic Score
Active, not recruiting; NCT02699047	Gastrointestinal cancer: effects of the fish oil intake on nutritional status, quality of life and immune and metabolic outcomes	I, R, DB	GI cancer, CRC, stomach cancer	Encapsulated fish oil vs. encapsulated olive oil	Change in quality of life, inflammatory response, body weight, body mass index, serum C-reactive protein, tumor markers (CEA, CA-19), serum albumin, survival and others
Active, not recruiting; NCT01661764	Fatty acid desaturase activity, fish oil and colorectal cancer prevention	I, R, DB	Adenomatous colorectal polyps	EPA and DHA vs. oleic acid	Rectal epithelial cell, proliferation, rectal epithelial cell apoptosis
Recruiting; NCT02179372	Modulation of fecal calprotectin by eicosapentaenoic free fatty acid in inflammatory bowel diseases	I, R, DB	UC, CD	EPA vs. MCFA (placebo)	Changes in FC levels
Completed; NCT02069561	Effects of eicosapentaenoic acid on molecular, metabonomics and intestinal microbiota changes, in subjects with long-standing inflammatory bowel disease	I, non-R, OP	UC	EPA vs. no intervention	Changes in RNA profiles, DNA methylation profiles, in cell proliferation and apoptosis
Completed; NCT02349594	Modulation of immune function by parenteral fish oil in patients with Crohn's disease and high inherent tumor necrosis factor-alpha production: a randomized, single blinded, cross-over study	I, R, SB	CD	Omegaven 10% vs. Intralipid 20%	Change in TNF-α production (pg/ml)

*CA-19, cancer antigen 19; CD, Crohn's disease; CEA, carcinoembryonic antigen; CRC, colorectal cancer; DHA, docosahexaenoic acid; EPA, eicosapentaenoic acid; FC, fecal calprotectin; GI, gastrointestinal; MCFA, medium chain fatty acid; UC, ulcerative colitis*.

*Study design: DB, double blinded; I, interventional; non-R, non randomized; OP, open label; R, randomized; SB, single blinded*.

## Conclusions

Polyunsaturated fatty acids (PUFAs) are an important group of bioactive lipids with pleiotropic effects in the organism. They affect immunity, inflammation and motility of the GI tract. Their usefulness was proven in FGIDs, in which PGE1-derived lubiprostone effectively alleviates the hallmark symptoms of IBS-C and CIC. In IBD and CRC, the potential of PUFAs is recognized but not fully utilized. When it comes to inflammation, the spotlight is currently on n-3 PUFAs, although AA-derived endocannabinoids also provide a promising target for pharmacological interventions. In CRC, the main focus is solely on n-3 PUFAs because of their inflammation- and immune-modulating properties. EPA and DHA are potentially useful as adjuvants to chemotherapy and possibly for CRC prevention in high-risk groups.

Metabolites of PUFAs also exert potent biological effects, more specific than FA themselves and thus should be of interest to pharmaceutical industry. EPA- and DHA-derived substances like Rvs or less known families of PGs could be useful as anti-inflammatory drugs. Finally, liposome-encapsulated n-3 PUFAs not only target specifically inflammatory sites e.g., IBD-related lesions or tumor mass, but can also be tracked in the human body. These modern drug delivery methods may be the key to unlocking true medical potential of PUFAs.

## Author contributions

PM and JF provided the overall concept and framework of the manuscript. AM and PM researched and identified appropriate articles, and participated in writing the manuscript. AM, PM, and JF revised the manuscript. All authors approved the final version of the manuscript.

### Conflict of interest statement

The authors declare that the research was conducted in the absence of any commercial or financial relationships that could be construed as a potential conflict of interest.

## Abbreviations

2-AG: 2-arachidonoylglycerol
12-LO: 12-lipooxygenase
5,6-EET: 5,6-epoxyeicosatrienoic acid
5-LO: 5-lipooxygenase
AA: arachidonic acid
AEA: anandamide
ALA: alpha-linoleic acid
CB: cannabinoid
CD: Crohn‘s disease
CIC: chronic idiopathic constipation
CLA: conjugated linoleic acid
CNS: central nervous system
COX: cyclooxygenase
CRC: colorectal cancer
CSLC: cancer stem like cells
CVD: cardiovascular disease
Cyp450: cytochrome 450
DHA: docosahexaenoic acid
DMPC: dimyristoylphosphatidylcholine
DP: prostaglandin D2 receptor
DPA: docosapentaenoic acid
DSS: dextran sulfate sodium
EETs: epoxyeicosatrienoic acids
ENS: enteric nervous system
EPA: eicosapentaenoic acid
EPA-FFA: free fatty acid eicosapentaenoic acid
EPIC: European Prospective Investigation of Cancer
FA: fatty acid
FAAH: fatty acid amide hydrolase
FAD1: fatty acid desaturase 1
FAP: familial adenomatous polyposis
FDA: Food and Drug Administration
FGIDs: functional gastrointestinal disorders
GI: gastrointestinal
GRAS: generally recognized as safe
HETEs: hydroxyeicosatetraenoic acids
HT-29-EP4: HT-29 human CRC cells with EP4 overexpression
IBD: inflammatory bowel disease
IBS: irritable bowel syndrome
IBS-A: alternating IBS
IBS-C: constipation-predominant IBS
IBS-D: diarrhea-predominant IBS
ICAM: intracellular adhesion molecule 1
iNOS: inducible nitric oxide synthase
LA: linoleic acid
LOX: lipooxygenase
LTB-4: leukotriene B-4
LXs: lipoxins
MPO: myeloperoxidase
NAFLD: non-alcoholic fatty liver disease
NO: nitric oxide
PBMC: peripheral blood mononuclear cells
PGA1: prostaglandin A1
PGD2: prostaglandin D2
PGE2: prostaglandin E2
PGH2: prostagandin H2
PGJ2: prostagandin J2
PGs: prostaglandins
PLA2: phospholipase A2
PUFAs: polyunsaturated fatty acids
RvD: D-type resolving
RvD1: resolvin D1
Rvs: resolvins
TNBS: 2,4,6-trinitrobenzene sulfonic acid
TRP: transient receptor potential
TRPA: TRP subfamily A receptor
TRPV4: transient receptor potential vanilloid type 4
TXs: thromboxanes
UC: ulcerative colitis
VCAM-1: vascular cell adhesion protein 1
VEGFR-2: vascular endothelial growth factor receptor 2.
